# Pseudohypoxic Brain Swelling After Elective Lumbar Spinal Surgery: Case Report

**DOI:** 10.7759/cureus.2454

**Published:** 2018-04-09

**Authors:** John Dickinson, Derek Kroll, Josh Bentley, Aaron J Gustin

**Affiliations:** 1 Neurological Surgery, Advocate Bromenn Medical Center; 2 Neurosurgery, Advocate Health Care

**Keywords:** spinal surgery, durotomy, cerebral spinal fluid, pseudohypoxic brain injury, pseudohypoxic brain swelling, venous cerebral congestion, postoperative intracranial hypotension-associated venous congestion, pihv, anoxic brain injury

## Abstract

Pseudohypoxic brain swelling (or the more recent term, postoperative intracranial hypotension-associated venous congestion) is a rare and potentially deadly complication that can occur after routine spine or brain surgery. The mechanism of this injury has been described as a rapid cerebral spinal fluid drainage leading to venous cerebral congestion. The clinical and radiographic findings mimic those found in a patient who has suffered an anoxic brain injury. We present the third reported case of postoperative intracranial hypotension-associated venous congestion following spinal surgery.

## Introduction

Pseudohypoxic brain swelling (PHBS), also more recently termed, postoperative intracranial hypotension-associated venous congestion (PIHV), is a rare and potentially fatal complication that can occur after uneventful spine or brain surgery. In 2003, Van Roost et al. first described PIHV in a study that reviewed 17 patients who postoperatively developed clinical features mimicking global cerebral hypoxia following the application of subgaleal suction drainage during cranial surgery [1]. In 2011, Parpaley et al. reported the first two cases of PIHV following spinal surgery with the application of epidural suction drainage [2]. To our knowledge, this is the third reported case of PIHV following spinal surgery.

## Case presentation

Our patient is a 71-year-old right-handed woman who developed acute low back and radicular pain. On further evaluation, she was found to have lumbar disc disease with herniation and associated spinal stenosis. She was admitted for intractable pain and taken to the operative theater for an elective decompressive laminectomy.

Prior to admission, she had maintained an active lifestyle without any limitations in her activities of daily living (ADL). Her past medical history included hypertension, dyslipidemia, hypothyroidism, osteopenia, and mild venous insufficiency. She is a non-smoker with no previous history of cerebral vascular accident or myocardial infarction.

Prior to this surgery, she had tolerated anesthesia well for several minor procedures without any previous complications. Of note, she had never previously received any spinal or cranial surgery.

She was taken to the operating room where she was carefully placed in the prone position. She underwent a posterior decompressive L3-L5 lumbar laminectomy, was found to have gross instability, and subsequently underwent pedicle screws and rod fixation of L3 to S1. The estimated blood loss (EBL) was 750 milliliters (mL), with a return of 280 mL via cell saver. Anesthetics and paralytics used during the case included fentanyl, Versed, desflurane, propofol, and rocuronium. A total of 4,500 mL of lactated ringers (LR) was given throughout the case. Total operative time was five hours. There was no cerebral spinal fluid (CSF) leak noted during the case. The case was uneventful with oxygen saturation at 97% or greater with normotensive blood pressures up to the time that the patient was receiving her last few subcutaneous sutures. Subfascial drains were then connected to full bulb suction, at which time she experienced a sudden, transient drop in systolic blood pressure, to the 50’s, that lasted approximately two minutes. She was rapidly turned over and appropriately resuscitated.

**Figure 1:**
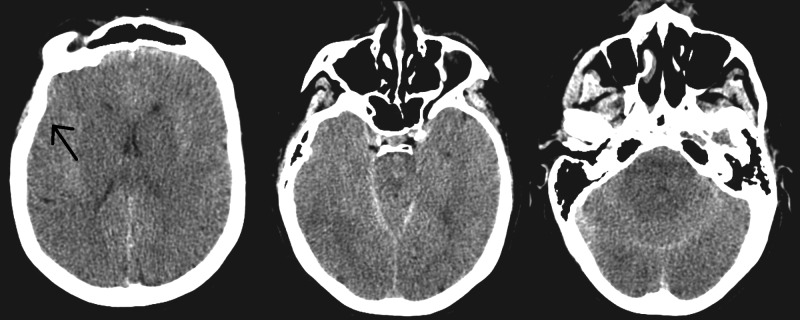
Postoperative day zero computed tomography of the head Diffuse cerebral edema, obliteration of cerebral of sulci and gyri is seen, consistent with an anoxic brain injury

After the case was completed, she had a delay in regaining consciousness so flumazenil and naloxone were administered; however, she remained unarousable. She was then taken for a computed tomography (CT) scan of the head that showed diffuse intracranial swelling consistent with anoxic brain injury (Figure 1).

**Figure 2:**
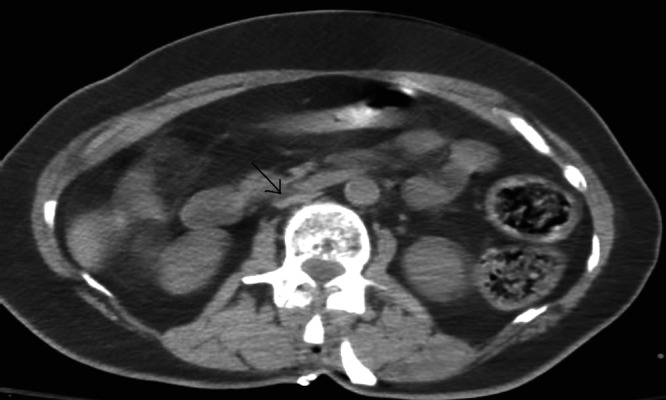
Flattened inferior vena cava

A CT scan of the abdomen was also performed to rule out any iatrogenic injury to the great vessels that could have occurred during pedicle screw placement. This study was found to be unremarkable, except that her inferior vena cava (IVC) appeared flat, indicative of decreased intravascular volume (Figure 2).

On examination, she had spontaneous respirations, no response to noxious stimuli, and absent vestibulo-ocular and corneal reflexes. She was transferred to the Neuro-Intensive Care Unit (ICU) and started on mannitol. Overnight, she experienced a single seizure episode without recurrence during the rest of her hospital course. Jackson-Pratt (JP) drains were discontinued on postoperative day 4 after being continued for high output (500 mL of fluid per day). High drain output of pink-tinged fluid, coupled with a stable hemoglobin, indicated the possibility of an occult intraoperative CSF leak. However, a sample of the pink-tinged fluid was never sent for beta-2-transferrin testing for confirmation.

**Figure 3:**
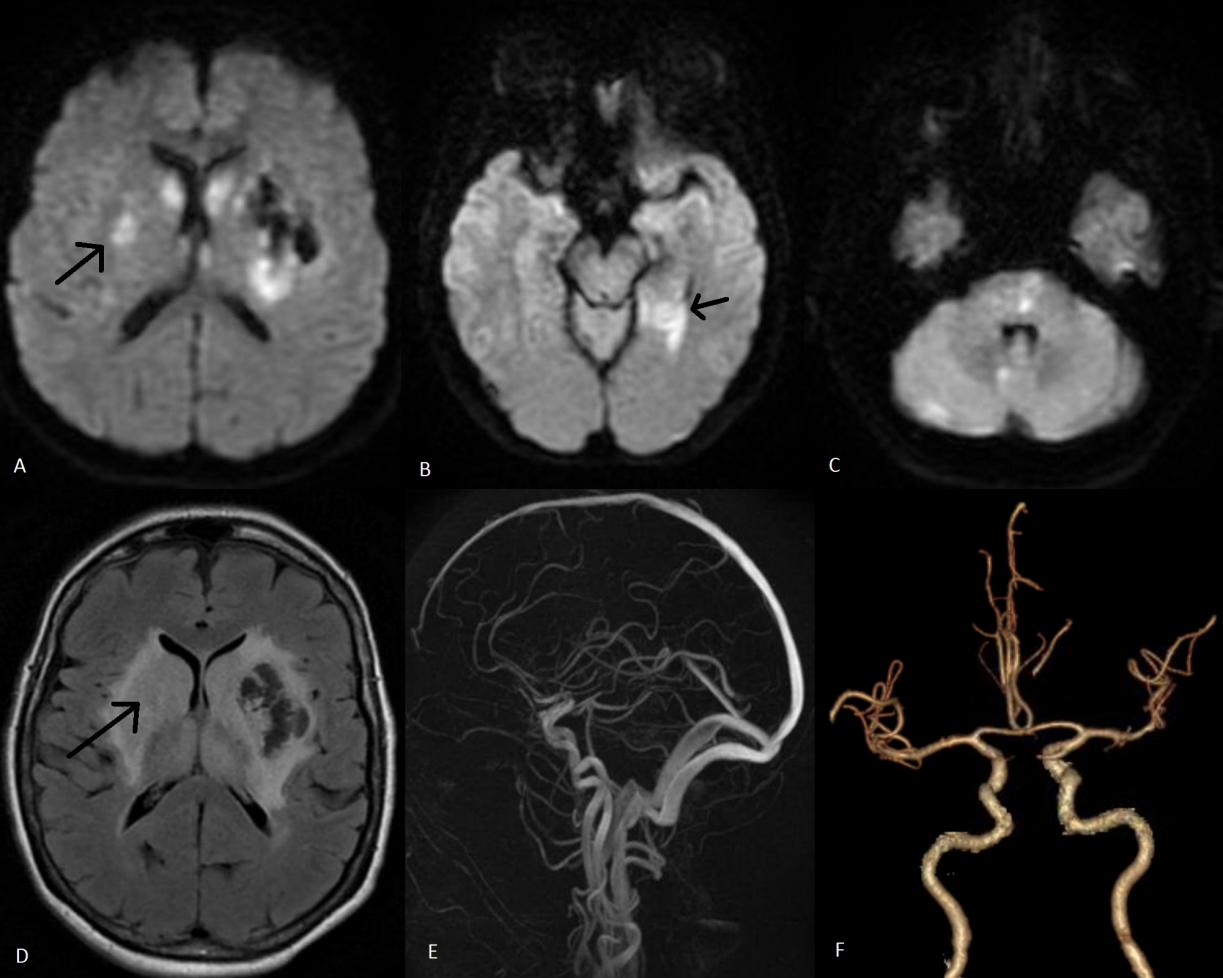
Magnetic resonance imaging brain and vascular studies done on postoperative day 5 (A-C) Diffusion-weighted magnetic resonance imaging shows multiple infarcts via patchy areas of diffusion restriction; (D) Fluid-attenuated inversion recovery signal changes in the bilateral basal ganglia; (E-F) Magnetic resonance venography and computed tomography angiography, respectively, show grossly normal vascular studies

On postoperative day 5, magnetic resonance imaging (MRI) of the brain was performed and revealed bilateral basal ganglion hemorrhages. CT angiography (CTA) of the head and magnetic resonance venography (MRV) were performed to rule out a thrombus or vascular insufficiencies that would predispose her to injury, both of which were found to be unremarkable. Nevertheless, MRI of the brain did show multiple infarcts, including supratentorial, infratentorial, and brainstem (Figure 3).

**Figure 4:**
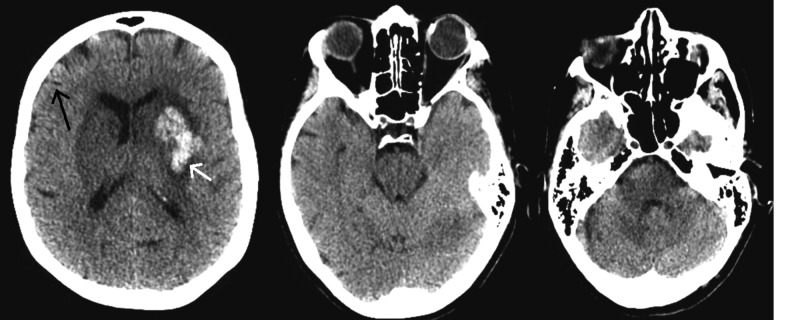
Computed tomography of the head done on postoperative day 8 Cerebral edema improves as gyri again become apparent (black arrow). New left basal ganglia hemorrhage (white arrow)

Serial CT head scans were performed throughout the hospital course, which showed gradual improvement of her cerebral edema in conjunction with a gradual, yet minor, improvement on clinical examination (Figure 4). She regained her pupillary and corneal reflexes, began opening her eyes, and withdrawing to noxious stimuli in all extremities.

By postoperative day 7, she began to follow simple commands. A tracheostomy and a percutaneous endoscopic gastrostomy (PEG) tube were placed on postoperative day 8. She was discharged from the hospital on postoperative day 9 to subacute rehabilitation. At one-year post-injury, she could communicate verbally and was able to ambulate with assistance.

## Discussion

The mechanism of cerebral venous congestion has been described using the Monro-Kellie doctrine. Once a subdural or subgaleal drain is set to suction in the presence of a CSF leak, known or unknown, there can be a rapid onset of intracranial hypotension. CSF hypovolemia creates a lack of volume that is replaced by blood or brain. Compensation should occur through an increase in cerebral blood volume (CBV), especially venous blood, as veins are more easily distended than arteries. Venous pooling then occurs and leads to congestion and subsequent venous infarction with concomitant cytotoxic and vasogenic edema [3].

We report the case of a 71-year-old female who underwent elective spine surgery and had an unexpected postoperative delay of consciousness with PIHV. Although likely underreported, it is too rare to believe that all CSF leaks with the placement of drains lead to this devastating complication.

In efforts to further reduce the incidence of PIHV and prevent this life-threatening complication, we hypothesize four risk factors that may contribute to cerebral venous congestion in these cases. 

1) Preoperative or intraoperative intravascular depletion: this can lead to the collapse of jugular veins, which then may cause a decrease in venous return and further exacerbate cerebral venous congestion.

2) Surgical positioning: certain positions can lead to pooling of blood intracranially and increase jugular venous pressure (JVP). If possible, place the patient in a slight reverse Trendelenburg position with the neck in a neutral position.

3) Watertight closure: following surgical repair of a CSF leak, Valsalva maneuver should be performed to check the integrity of the repair and ensure that a watertight closure has been achieved.

4) The velocity of CSF depletion: it has been reported that when subgaleal/subfascial drains are placed to suction, it is the velocity of CSF depletion that has been shown to induce PIVH and not necessarily the volume [4]. In the setting of CSF leaks, consider thumbprint or no suction.

To date, there are no definitive treatment options for the reversal of PIHV. However, in 2002, Binder et al. presented a patient that became obtunded secondary to spontaneous intracranial hypotension [5]. In order to normalize the intracranial pressure, they performed an intrathecal saline infusion. The patient quickly regained consciousness once the intracranial pressure had normalized. Therefore, an increase in intrathecal volume may lead to a decrease in venous congestion and possibly improve clinical outcomes. Nevertheless, this treatment has not yet been tested in patients with PIHV and should be considered experimental and only implemented in those patients that have failed to show clinical improvement after all other management options have been attempted.

## Conclusions

PIHV is a rare, yet devastating potential complication of any cranial or spinal procedure. Aside from avoiding suction drains in the presence of a spinal fluid leak, there has been little discussed on how to avoid this complication and even less on how to reverse it. Avoidance of preoperative and intraoperative intravascular depletion, careful surgical positioning, the performance of an intraoperative Valsalva maneuver, and placement of subgaleal/subfascial drains to thumbprint suction or no suction are suggested ways that may lower the incidence of this newly recognized complication. Further, we theorize that an intrathecal saline infusion, an untested treatment option in this population, should be given consideration after all other management options have been exhausted.
